# Pre-pregnancy evaluation of essential hypertensive women with poor obstetric history

**DOI:** 10.1590/1806-9282.20251913

**Published:** 2026-06-29

**Authors:** Murat Cagan, Hanife Guler Donmez, Erdem Fadiloglu, Gonca Ozten, Mehmet Sinan Beksac

**Affiliations:** 1 Hacettepe University, Faculty of Medicine, Department of Obstetrics and Gynecology – Ankara, Türkiye.; 2 Hacettepe University, Faculty of Science, Department of Biology – Ankara, Türkiye.; 3 İstinye University, Liv Hospital Ankara, Department of Obstetrics and Gynecology – Istanbul, Türkiye.

**Keywords:** Preconception care, Essential hypertension, Inflammation, Glucose metabolism disorders, Gestational outcome

## Abstract

**OBJECTIVE:**

Essential hypertension is linked to increased maternal and perinatal risks. The aim of this study was to assess women with essential hypertension and poor obstetric history before conception to identify co-morbidities and placenta-related risk factors and to examine how preconception evaluation affects future pregnancy outcomes.

**METHODS:**

This retrospective cohort study involved women with poor obstetric history, with (n=25) or without (n=243) essential hypertension. Women were evaluated for co-morbidities and metabolic, immunological, inflammatory, and vascular/thrombotic risk factors. Subsequent pregnancies were then assessed for gestational outcomes.

**RESULTS:**

Higher levels of co-morbidity were found in the essential hypertension (+) group compared to high-risk controls (68 vs. 39.9%, p=0.013). Essential hypertension (+) women had significantly higher rates of chronic inflammatory diseases (28 vs. 5.8%, p=0.001) and carbohydrate metabolism disorders (44 vs. 10.3%, p<0.001) than in controls. Except for “APGAR<7” (p=0.016), we were unable to show any statistically significant differences between the study and control groups in terms of placenta-related obstetric complications and gestational outcomes in their forthcoming pregnancies due to necessary precautions and timely management of risk factors.

**CONCLUSION:**

Chronic inflammatory diseases and carbohydrate metabolism disorders, which are risk factors for both endothelial injury and placenta-related obstetric complications, are more frequently observed in pregnancies with essential hypertension. Preconception counseling is critical in reducing gestational complications.

## INTRODUCTION

Essential hypertension (EH) is present in 0.9–2% of pregnant women, and the rate is increasing mainly secondary to the obesity epidemic and advanced maternal age^1,2^. Reproductive-age women with EH may have no symptoms, but high pressure may harm the vessels and cause clinical problems. A wide range of healthcare professionals are concerned about pregnant women with EH because they are more likely to experience maternal and perinatal complications^2^.

Endothelial cell injury and inflammation play a crucial role in the pathogenesis of endothelial dysfunction and EH^3,4,5^. The endothelium is an essential metabolizing and endocrine organ that controls blood fluidity, vascular tone, cellular growth, and leukocytes/thrombocytes-vessel wall interaction. Additionally, it produces thrombo-regulatory molecules and growth factors and reacts to chemical and physical signals^3,4^. On the other hand, “gestational hypertension/preeclampsia” is defined as the presence of hypertension, edema, and proteinuria during the course of pregnancy, and it is one of the main reasons for adverse pregnancy outcomes and poor perinatal morbidity/mortality^6,7^. Preeclampsia (PE) is a placenta-related obstetric complication (PROC) with a broad spectrum of pathological events^6,7^. Immunological, metabolic, and chronic inflammatory factors may damage the cellular components of the intervillous space, and the resultant placental inflammation may induce obstetrical complications, including hypertensive disorders of pregnancy^8^.

Hypertensive disorders of pregnancy complicate about 10% of pregnancies, resulting in increased maternal and perinatal morbidity/mortality^9,10^. Diagnosis of hypertension in reproductive-age women is uneasy, with many disorders often remaining unrecognized and therefore poorly managed before and during pregnancy^9^. Besides, co-existing systemic disorders and risk factors for gestational hypertension/PE were sometimes ignored due to various reasons, though they are very important in the personalized management of hypertensive disorders of pregnancy^1,2,9,10^. Here comes the necessity of pre-pregnancy evaluation and preconception counseling for women with EH^8,11,12^. Preconception counseling/care is critical in decreasing the impact of medical, behavioral, and social risks on maternal and fetal health.

There is an ongoing debate on whether or not pregnant women with EH should routinely use antihypertensive drugs. Recommendations were inconsistent in the management of EH across widely accepted guidelines, and even the rates of the superimposition of gestational hypertension and PE are still to be clarified^1,10,11,12^. Thus, personalized management of EH in pregnancy seems to be more realistic in clinical practice^2^.

This retrospective study aimed to show the significance and the beneficial effect of the pre-conception evaluation of women with poor obstetric history with or without EH (in terms of the presence of “co-morbidities/risk factors” for PROCs and adverse gestational outcome) on forthcoming gestations.

## METHODS

This was a retrospective descriptive study comprising two groups of patients with poor obstetric history: (1) Women with EH (n=25) and (2) Women without EH (n=243). Within the framework of a unique pre-pregnancy care program, all patients were admitted to preconception counseling between January 2016 and January 2020. The study was conducted in accordance with the Declaration of Helsinki, and ethical approval (GO19/1064) for the conduct of this study was granted by the Local Ethics Committee of our Institution. Necessary laboratory tests were performed together with the physical/gynecological examinations. EH is defined as a disorder characterized by a pathological increase in blood pressure, indicated by a persistent elevation in blood pressure exceeding 140 over 90 mm Hg. Currently, EH is defined when the systolic pressure is consistently greater than 140 mm Hg or when the diastolic pressure is consistently 90 mm Hg or higher^1,2^. In this study, Nifedipine was preferred in a personalized manner in necessary cases to treat high blood pressure. Cases whose blood pressures were not under control were not advised to get pregnant until they had normal blood pressures. The efficacy of Nifedipine in reducing blood pressure and inhibiting threatened preterm contractions is well established, and this is the reason why this drug is selected for the patients of this specific cohort^13,14^.

All women were evaluated before conception, and their assessment included an evaluation for relevant co-morbidities, as well as risk factors associated with PROCs, adverse gestational outcomes, and EH. Adverse gestational outcome was defined as the presence of at least one of these outcomes: miscarriage, preterm births, stillbirth, APGAR score <7, and necessity of neonatal intensive care unit (NICU). Risk factors specifically selected for this study were as follows: Autoimmune disorders, chronic inflammatory diseases, metabolic disorders, and thrombotic events. PROCs were defined as miscarriage, fetal growth restriction, PE, and preterm birth.

After confirming their pregnancies, patients with risk factors for PROCs were prescribed a prophylactic regimen. This included low-dose low-molecular-weight heparin (LMWH) (enoxaparin 2000 anti-Xa IU/0.2 mL), low-dose salicylic acid (100 mg acetylsalicylic acid), and low-dose corticosteroid (methylprednisolone, 1×4 mg/day orally in necessary cases)^8^. Serial ultrasonography was used to monitor fetal growth during pregnancy, as well as aneuploidy screening, fetal anatomy scanning, an oral glucose challenge test, routine blood tests, and a non-stress test that was done once a week after week 28 of gestation.

Demographic characteristics, risk factors for PROCs, gestational age at birth, birth weight, APGAR scores, NICU admissions, and Beksac Obstetric Index (BOI) were compared between the control and study groups. The BOI, calculated as [number of living children+(π/10)]/gravida, and its pregnancy-specific value (BOIp) were examined. Data for all study participants who gave birth at University Hospital between 2016 and 2020, were extracted from the electronic database of the Division of Perinatology.

Demographic data and BOIp were presented as numbers, percentages, and mean±standard deviation (SD). The Shapiro-Wilk test was used to analyze normality. Normally distributed data were compared by an independent t-test. The Mann-Whitney U test was used for comparison of two groups non-parametrically. For categorical data, the chi-square test or Fischer’s exact test was used for the comparison. Asterisks shown in the tables indicate significant differences between groups (*p<0.05). The Statistical Package for the Social Sciences Software version 23 (SPSS Inc., Chicago, IL) was used for statistical analysis.

## RESULTS

A total of 268 women with poor obstetric history (25 EH [+] and 243 EH [-]) were included in the present study. Table 1 shows the comparison of clinical and demographic findings. Both study and control groups were comparable in terms of maternal age, gravidity, parity, number of miscarriages, and BOIp (p>0.05 for all).

**Table 1:** The comparison of control and study groups in terms of maternal age, gravidity, parity, miscarriage, and Beksac Obstetric Index during pregnancy.

	EH (-) n=243	EH (+) n=25	Total n=268	p
Maternal age				
Mean±SD	33.1±5.11	34.2±4.98	33.2±5.10	0.319^a^
Gravidity				
Mean±SD	4.2±1.95	4.1±1.93	4.2±1.94	0.702^b^
Parity				
Mean±SD	1.6±1.04	1.8±1.09	1.7±1.04	0.329^b^
Miscarriage				
Mean±SD	1.9±1.89	2.0±2.30	1.9±1.93	0.886^b^
BOIp				
Mean±SD	0.551±0.31	0.614±0.33	0.557±0.32	0.276^b^

^a^Independent t-test;

^b^Mann-Whitney U test; EH: essential hypertension; BOIp: Beksac Obstetric Index during pregnancy; SD: standard deviation.

Table 2 shows the rates of maternal risk factors for PROCs. Notably, higher levels of co-morbidity were found in the EH (+) group compared to controls (68 vs. 39.9%, p=0.013). Chronic inflammatory diseases and carbohydrate metabolism disorders are leading comorbid conditions. EH (+) women had significantly higher rates of chronic inflammatory diseases (28 vs. 5.8%, p=0.001) and carbohydrate metabolism disorders (44 vs. 10.3%, p<0.001) than in controls. Both the EH (+) and control groups showed similar rates of immune system problems, methylenetetrahydrofolate reductase (MTHFR) polymorphisms, and hereditary thrombophilia (p>0.05 for all).

**Table 2:** Evaluation of control and study groups in terms of the presence of maternal risk factors for placenta-related obstetric complications.

Maternal risk factors	EH (-) n=243	EH (+) n=25	Total n=268	p
Co-morbidity (-)	146 (60.1)	8 (32.0)	154 (57.5)	0.013^a *^
Co-morbidity (+)	97 (39.9)	17 (68.0)	114 (42.5)	
Immune system problems (-)	216 (88.9)	19 (76.0)	235 (87.7)	0.100^b^
Immune system problems (+)	27 (11.1)	6 (24.0)	33 (12.3)	
Chronic inflammatory diseases (-)	229 (94.2)	18 (72.0)	247 (92.2)	0.001^b *^
Chronic inflammatory diseases (+)	14 (5.8)	7 (28.0)	21 (7.8)	
MTHFR polymorphism (-)	131 (53.9)	18 (72.0)	149 (55.6)	0.128^a^
MTHFR polymorphism (+)	112 (46.1)	7 (28.0)	119 (44.4)	
Hereditary thrombophilia (-)	190 (78.2)	22 (88.0)	212 (79.1)	0.373^b^
Hereditary thrombophilia (+)	53 (21.8)	3 (12.0)	56 (20.9)	
Carbohydrate metabolism problems (-)	218 (89.7)	14 (56.0)	232 (86.6)	<0.001^b *^
Carbohydrate metabolism problems (+)	25 (10.3)	11 (44.0)	36 (13.4)	
Total	25 (100)	243 (100)	268 (100)	

^a^Yates continuity test;

^b^Fisher’s exact test; ET: essential hypertension;

^*^p<0.05, Maternal risk factors are shown as percentages per subgroup (in parentheses) and numbers. Comorbidity is defined as the presence of at least one of the maternal risk factors. MTHFR: methylenetetrahydrofolate reductase.

The adverse gestational outcome is defined as the presence of at least one of these outcomes, including miscarriage, preterm births, stillbirth, APGAR score <7, and the necessity of a NICU. As seen in Table 3, the rates of adverse pregnancy outcomes were 48 and 43.2% in the EH (+) and control groups, respectively. Preterm birth, PE, and stillbirth rates were higher in the EH (+) group than in the controls, except for miscarriage, although there was no statistically significant difference between the groups (p>0.05 for all). EH (+) and control groups were comparable in terms of birthweight, gestational day at birth, and NICU admission (p>0.05 for all); however, an “APGAR<7” was found more frequently in neonates of EH (+) women (p=0.016).

**Table 3:** The comparison of the control and study groups in terms of placenta-related obstetric complications and gestational outcome.

Placenta related obstetric complications	EH (-)	EH (+)	Total	p
Adverse gestational outcome (-)	138 (56.8)	13 (52.0)	151 (56.3)	0.804^a^
Adverse gestational outcome (+)	105 (43.2)	12 (48.0)	117 (43.7)	
Preterm birth				
(-)	227 (93.4)	22 (88.0)	249 (92.9)	0.400^b^
(+)	16 (6.6)	3 (12.0)	19 (7.1)	
Preeclampsia				
(-)	240 (98.8)	24 (96.0)	264 (98.5)	0.326^b^
(+)	3 (1.2)	1 (4.0)	4 (1.5)	
Miscarriage				
(-)	183 (75.3)	22 (88.0)	205 (76.5)	0.239^a^
(+)	60 (24.7)	3 (12.0)	63 (23.5)	
Stillbirth				
(-)	242 (99.6)	24 (96.0)	266 (99.3)	0.178^b^
(+)	1 (0.4)	1 (4.0)	2 (0.7)	
APGAR score				
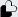 7	171 (96.1)	16 (80.0)	187 (94.4)	0.016^b *^
<7	7 (3.9)	4 (20.0)	11 (5.6)	
Total				
Neonatal Intensive Care Unit				
(-)	144 (80.9)	14 (70.0)	158 (79.8)	0.249^b^
(+)	34 (19.1)	6 (30.0)	40 (20.2)	
Birthweight (g)				
Mean±SD	2,929.83±422.7	2,954.00±549.8	2,932.27±435.6	0.433^c^
Gestational age at birth (day)				
Mean±SD	258.79±9.06	255.55±15.11	258.46±9.83	0.293^c^

^a^Yates continuity test;

^b^Fishers’ exact test;

^c^Mann-Whitney U test; EH: essential hypertension; SD: standard deviation. Placenta-related obstetric complications and adverse pregnancy outcomes are shown as percentages per subgroup (in parentheses) and numbers. “Adverse gestational outcome” is defined as the presence of at least one of these outcomes: miscarriage, preterm births, stillbirth, APGAR score <7 and necessity of neonatal intensive care unit (NICU)

^*^p<0.05.

## DISCUSSION

In women with EH, the blood pressure may remain stable in early pregnancy and may increase sometimes during the second or third trimester. The frequency with which severe accelerated hypertension occurs is controversial, and studies have suggested a rate of 2–11%^15^. Thus, it is critical to specify/predict hypertensive women who will end up with accelerated EH and/or superimposition of gestational hypertension/PE. Besides, the rate of the occurrence of PE was reported to be 48% in women with chronic hypertension^16^. At this point, we believe that we should focus on the co-morbidities associated with the injury of endothelial tissue and inflammation, which are risk factors for both EH and PROCs. In this study, we have demonstrated statistically significantly increased rates of chronic inflammatory diseases and carbohydrate metabolism disorders in women with EH compared to control group patients who also had poor obstetric history and various types of risk factors. Consistent with our results, carbohydrate metabolism abnormalities and inflammatory diseases are reported to play a role in the etiology and clinical progression of PE^17,18^. Fortunately, we were unable to show any statistically significant differences between the study and high-risk control groups in terms of PROCs and gestational outcomes with the exception of “APGAR<7” (p=0.016), most probably due to timely management of co-morbidities and risk factors. Here comes the beneficial effect of pre-pregnancy evaluations to take necessary precautions for women with poor obstetric history and various types of co-morbidities.

The overall estimates of PE were reported to be 4.6% with a wide variation across regions, which may be the result of the injury of the cellular components of the intervillous space and the resultant placental inflammation^19^. In this series, we did not observe PE or acceleration of hypertension in pregnancies with EH, most probably due to pre-pregnancy precautions/management and the prophylactic use of low-dose LMWH plus low-dose acetylsalicylic acid (and low-dose corticosteroid in necessary cases) during antenatal care^8^. Low-dose acetylsalicylic acid (at dosages of 81–100 mg) initiates the biosynthesis of anti-inflammatory mediators (15-epi-Lipoxin 4A), which are distinct from the anti-platelet activity of aspirin. Plasma 15-epi-Lipoxin 4A levels significantly increased in the 81-mg aspirin cases, with borderline increases in the 325-mg cases and no apparent significant changes in the 650-mg cases. When 15-epi-Lipoxin A4 and Thromboxane B2 (anti-platelet activity) were compared, levels changed in a statistically significant and opposite direction for all three aspirin doses^20^. On the other hand, LMWH may convey beneficial effects in placenta-related complications independent of their anticoagulant activity, possibly by inhibiting placental inflammation (anti-inflammatory effect). LMWH activates the PI3-kinase-AKT pathway via heparin-binding epidermal growth factor signaling in trophoblasts/macrophages, thus preventing inflammasome activation. Moreover, LMWH inhibits NLRP3 (NLR family pyrin domain-containing 3) inflammasome activation^21^. These findings also accentuated the importance of pre-conception counseling and careful/detailed antenatal care of pregnant women with poor obstetric history with or without EH.

It is difficult to make a diagnosis of EH in a woman whose elevated blood pressure is being evaluated for the first time during pregnancy. The diagnosis can be made most confidently when the hypertension has been noted before pregnancy^22^. We believe that pre-conceptional evaluation of women with poor obstetric history and EH is critical to demonstrate co-morbidities and risk factors for both accelerations of EH and obstetrical complications in order to have necessary precautions and better management protocols.

The primary limitations of this study are the retrospective design and the high-risk character of the control group. The relatively small number of women in the EH group and the study’s single-center design may also be considered as additional limitations. In this study, Nifedipine was chosen as the drug of choice (in a personalized manner) in necessary cases to treat high blood pressure. However, we could not search the effect of antihypertensive drug usage on gestational outcome due to the study design and the population characteristics of the patients with EH. Pharmacologic management of hypertension throughout gestation has undoubtedly been one important issue in the reduction of the frequency of accelerated hypertension in pregnancy^23^. However, the advantages of lowering blood pressure during pregnancy (especially in mild and moderate cases) and the ideal blood pressure targets are still up for debate^24^. It has also been reported that the treatment of hypertension in pregnancy does not appear to alter disease progression in PE, especially in mild to moderate cases^25^.

## CONCLUSION

In this study, chronic inflammatory diseases and carbohydrate metabolism disorders, which are risk factors for both endothelial injury and PROC, are more frequently observed in pregnancies with EH. In conclusion, pre-pregnancy evaluation of women with poor obstetric history and EH is important in reducing gestational complications.

## Data Availability

The datasets generated and/or analyzed during the current study are available from the corresponding author upon reasonable request.
